# An Accurate GNSS Spoofing Detection Method Based on Multiscale Eye Diagrams

**DOI:** 10.3390/s25030903

**Published:** 2025-02-02

**Authors:** Chuanyu Wu, Yuanfa Ji, Xiyan Sun

**Affiliations:** 1 Information and Communication School, Guilin University of Electronic Technology, Guilin 541004, China; wcy@mails.guet.edu.cn; 2 Guangxi Key Laboratory of Precision Navigation Technology and Application, Guilin University of Electronic Technology, Guilin 541004, China; 3 International Joint Research Laboratory of Spatio-Temporal Information and Intelligent Location Services, Guilin University of Electronic Technology, Guilin 541004, China; 4 GUET-Nanning E-Tech Research Institute Co., Ltd., Nanning 530031, China

**Keywords:** GNSS spoofing detection, eye diagram detection method, multiscale Canny algorithm, minimum misjudgment probability

## Abstract

Spoofing detection is critical for GNSS security. To address the issues of low detection rates and insufficient coverage in traditional methods, this study proposes an eye diagram detection method based on the multiscale Canny algorithm with minimum misjudgment probability (EDDM-MSC-MMP). Unlike conventional correlation peak distortion detection techniques, the proposed method uses the MSC-MMP algorithm to perform multiscale edge extraction from the eye diagram generated from the receiver’s correlation values. It then calculates the image threshold using minimum misjudgment probability to ensure the accuracy of the eye diagram’s edges. This enables the accurate detection of subtle changes in the eye diagram, leading to the better identification of spoofing signals. The results show that the MSC-MMP outperforms traditional edge extraction algorithms by over 0.072 in terms of the optimal dataset scale F score (ODS-F). Compared to signal quality monitoring (SQM) and Carrier-to-Noise Ratio methods, the EDDM-MSC-MMP method increases spoofing detection coverage by over 60%, achieving the highest detection rate in the TEXBAT dataset. Overall, the EDDM-MSC-MMP method improves the reliability and coverage of spoofing detection, providing an effective solution for GNSS spoofing detection.

## 1. Introduction

Civilian global navigation satellite system (GNSS) frequencies are accessible to the general public and provide services for navigation, positioning, and timing under all weather conditions on a global scale [1]. These systems are becoming increasingly important in contemporary society. In the past, the primary concerns of users and developers were the availability and accuracy of these systems. However, with the advancement of this technology and the expansion of its application areas, security issues have begun to attract widespread attention and have become a key factor in improving system performance [2,3]. The inherent weakness of GNSS signals renders them susceptible to radio frequency interference [4], particularly spoofing interference. This type of interference is particularly insidious because it can result in the transmission of spoofed GNSS signals, which can cause the receiver to output incorrect position, velocity, and time (PVT) information without the user’s knowledge [5,6,7], thereby seriously compromising user safety. It is therefore critical to improve the ability of GNSS receivers to detect spoofed signals to ensure user safety.

To safeguard satellite signals, several spoofing detection methods have been devised. At the level of signal transmission, Humphreys proposed a cryptographic authentication technique designed to thwart spoofing attacks [8]. This approach involves encrypting navigation signals and verifying the accuracy and integrity of the received signals. However, this cryptography-based detection method necessitates alterations to the satellite signal source, which is prohibitively expensive and challenging to implement in the near future, particularly in light of the constraints inherent to the existing signal system architecture.

Research at the signal reception end has concentrated on analyzing the anomalous signal characteristics resulting from spoofing attacks. These studies can be classified into three categories. The first category employs spatial processing techniques utilizing a receiver antenna array [9,10,11], and these techniques have robust antispoofing capabilities. However, this approach is costly and necessitates the use of expensive antenna hardware. The second category comprises baseband signal processing techniques that operate within the receiver’s tracking loop [12], including signal quality monitoring (SQM) [13], Doppler anomaly detection [14], and signal energy detection [15,16,17]. The third category includes the postprocessing of observational data after the receiver has been located [18]; such methods perform consistency checks on multiple sets of disparate data, such as ephemeris data and clock offset data detection.

Among the abovementioned detection methods, the second type of signal reception processing technology is favored by many researchers because it does not require additional hardware, has low complexity and cost, and is easy to implement. In particular, the SQM metrics, a series of detection methods based on the output value of the correlator, which include Delta, Ratio, Slope, and ELP, can all be implemented in tracking the receiver [19,20]. Phelts first proposed the two SQM metrics Delta and Ratio, which aim to identify signal abnormalities by detecting changes in the correlation peak function [21]. The Delta metric is mainly used to determine the symmetry on both sides of the correlation peak function, whereas the Ratio metric is used to evaluate the distortion at the top of the correlation peak function. However, when the code phase, carrier phase, and $C/N_{0}$ are very close to those of the real signal, the change in the correlation peak may not be obvious [22], which greatly reduces the effectiveness of detection. Mubarak proposed the use of the early-late phase (ELP) correlator of the tracking loop to identify spoofing signals [23]. Although the ELP can accurately detect small code phase differences, its detection performance significantly decreases when the carrier phase of the spoofing signal is close to an integral multiple of the true signal, in which case, the probability of detecting the spoofing signal is reduced almost to zero. Khan determined the presence of spoofing signals by comparing the estimated slope of the correlation peak function with its expected value [13]. This method requires an additional correlator, and its effectiveness is strongly impacted by the position of the correlator. When the distortion of the correlator position is large, the detection performance is excellent, but, when the distortion is small, the effect is not good. Wang and Wesson conducted a comparative analysis of the Slope metric against other metrics and validated their findings using the Texas Spoofing Test Battery (TEXBAT) dataset from the University of Texas [16,24]. Ali used a combination of two metrics to comprehensively evaluate the quality of the autocorrelation function [25]. Jahromi introduced $C/N_{0}$ as a parameter for detecting spoofing signals [26], which we refer to as the CN0 method hereafter. This method results in greatly reduced detection performance when the signal strength is low or when the spoofing signal increases the signal power and noise simultaneously.

The eye diagram is a common tool used in the field of communications to assess the performance of digital signal transmission systems. It visually displays the time-domain characteristics and amplitude characteristics of the signal, especially the distortions and noise effects during signal transmission. The edge features of the eye diagram, such as the eye height, are a key part of understanding and analyzing signal integrity. This paper presents an eye diagram detection method based on a multiscale Canny algorithm with minimum misjudgment probability (EDDM-MSC-MMP). This approach aims to enhance the edge detection capability of the eye diagram and improve the detection of spoofing signals through eye diagram analysis. The method generates an eye diagram by extracting the pertinent values from the receiver and determines whether a spoofing signal is present by extracting the eye height and eye width data from the eye diagram via the multiscale Canny algorithm with minimum misjudgment probability (MSC-MMP). Compared with CN0-based methodologies, the EDDM-MSC-MMP exhibits superior detection efficacy in the context of spoofing attacks that do not alter the $C/N_{0}$ value. During such attacks, the overall signal strength tends to increase, yet the $C/N_{0}$ value may remain unaltered, thereby rendering CN0-based detection methods ineffective. The EDDM-MSC-MMP is capable of fully extracting the signal strength, effectively addressing scenarios in which the carrier power and noise power increase simultaneously, and overcoming the detection limitations of the CN0 method. In contrast to the signal quality monitoring method, the EDDM-MSC-MMP method does not rely on the distortion of the relevant peak function but rather directly observes the specific changes in the correlation value eye diagram to detect spoofing signals more effectively.

The specific contributions of this paper are summarized as follows:

1. We review the signal model of the receiver, explain the differences between real signals and spoofed signals, and discuss the impact of spoofing attacks on the signal.
2. We propose the eye diagram detection method based on a multiscale Canny algorithm with minimum misjudgment probability (EDDM-MSC-MMP). This method addresses the issue of low detection rates, or even the inability to detect spoofing attacks effectively, in certain spoofing scenarios encountered by traditional SQM and CN0 methods, providing a solution for spoofing detection.
3. We introduce the proposed MSC-MMP algorithm, which effectively suppresses interference from texture edges while maintaining edge integrity. This algorithm solves the problem of capturing fine texture edges and inaccurate edge localization when using large Gaussian windows while also avoiding the drawback of retaining excessive texture edges when using small-scale windows. We conduct a thorough comparison between the proposed MSC-MMP algorithm and traditional edge detection techniques (Log, Prewitt, Sobel, Zero Crossing, and Canny) using a custom-built eye diagram dataset. The results demonstrate that the MSC-MMP outperforms these methods in terms of edge detection accuracy and robustness.
4. Performance evaluation in spoofing detection coverage: Through extensive simulation experiments, we evaluate the performance of the EDDM-MSC-MMP method in detecting spoofing attacks under various conditions, including power, code phase offset, and carrier offset. Our results show a significant improvement in the detection rate and coverage compared to the existing SQM metrics and CN0 method.
5. Real-data validation using the TEXBAT dataset: We validate our method using the TEXBAT dataset, which contains real-world spoofing signals collected by the University of Texas. The experimental results confirm that the EDDM-MSC-MMP exhibits better performance in detecting spoofing attacks, with a superior ROC curve and a higher detection rate compared to existing methods.

## 2. Signal Models

In a typical GPS receiver, the received signal model and the correlation signal model are key to understanding the behavior of satellite signal transmission. By analyzing the characteristics of the correlation signal model, an appropriate spoofing detection strategy can be formulated. This section introduces these two signal models.

### 2.1. Received Signal Model

The signals received from a satellite are frequently affected by many types of interference. In particular, when spoofing signals are present, they are often a significant source of error in signal processing. For example, consider the GPS L1 signal. When spoofing signals are present, the received signal model, after processing by the RF front end, can be expressed as follows [27]:

(1) $$\begin{array}{l} S\left(t\right)\\ \;\;\;=S_{a}\left(t\right)+S_{s}\left(t\right)+n\left(t\right)\\ \;\;\;=\sqrt{2P_{a}}C_{a}\left(t-\tau \right)D_{a}\left(t-\tau \right)\cos\left(2\pi f_{a}\left(t-\tau \right)+\theta_{a}\right)\\ \;\;\;\;\;\;+\sqrt{2P_{s}}C_{s}\left(t-\tau -\Delta \tau_{s}\right)D_{s}\left(t-\tau -\Delta \tau_{s}\right)\cos\left(2\pi \left(f_{a}+\Delta f_{s}\right)\left(t-\tau -\Delta \tau_{s}\right)+\theta_{a}+\Delta \theta_{s}\right)+n\left(t\right) \end{array}$$

where $S\left(t\right)$ is the intermediate frequency signal received at time t; $S_{a}\left(t\right)$ and $S_{s}\left(t\right)$ are the real signal and spoofing signal, respectively; $n\left(t\right)$ is additive Gaussian white noise with mean 0, power spectral density $N_{0}/2$, and variance $\delta^{2}$; $P_{a}$ and $P_{s}$ are the average powers of the real signal and spoofing signal, respectively; $C_{a}$ and $C_{s}$ are the C/A codes of the real signal and spoofing signal, respectively; $D_{a}$ and $D_{s}$ are the data codes of the real signal and the spoofing signal, respectively; $f_{a}$ is the carrier frequency of the real signal; $\Delta f_{s}$ is the difference in carrier frequency between the spoofing signal and the real signal; $\theta_{a}$ is the initial carrier phase of the real signal; $\Delta \theta_{s}$ is the difference in the initial carrier phases of the spoofing signal and the real signal; τ is the propagation delay of the real signal; and $\Delta \tau_{s}$ is the difference in the propagation delays of the spoofing signal and the real signal.

Equation (1) shows that the received signal is a superposition of the real signal, the spoofing signal, and Gaussian white noise. The spoofing signal resembles the real signal in terms of its structural composition; however, there are notable discrepancies between the two, particularly with respect to their respective signal power levels, code delays, Doppler frequencies, initial carrier phases, and navigation messages. These differences enable the real and spoofed signals to be identified and distinguished.

### 2.2. Correlator Estimation Model

In the signal tracking stage, the received intermediate frequency signal is subjected to a series of operations, including carrier stripping and correlation integration, before being converted into a baseband signal comprising the data code alone. In the absence of a spoofing signal, the in-phase channel (I-channel) and quadrature channel (Q-channel) signals can be expressed as follows:

(2) $$\left\{\begin{array}{c} I_{d}=\sqrt{2P_{a}}D\left(t\right)R\left(\tau \right)\cos\left(\omega_{e}t+\theta_{e}\right)+n_{i}\left(t\right)\\ Q_{d}=\sqrt{2P_{a}}D\left(t\right)R\left(\tau \right)\sin\left(\omega_{e}t+\theta_{e}\right)+n_{q}\left(t\right) \end{array}\right.$$

where $I_{d}$ and $Q_{d}$ are the signal output results of the I-channel and Q-channel, respectively; $\omega_{e}$ and $\theta_{e}$ are the carrier frequency difference and initial phase difference between the true signal and the receiver’s copy, respectively; $P_{a}$ is the power of the real satellite signal; $\omega_{e}=2\pi \left(f_{a}-f_{0}\right)$; $\theta_{e}=\theta_{a}-\theta_{0}$; $f_{0}$ and $\theta_{0}$ are the frequency and initial phase of the copy carrier, respectively; $R\left(\tau \right)$ is the normalized autocorrelation function; and the Gaussian noise terms $n_{i}\left(t\right)$ and $n_{q}\left(t\right)$ correspond to the I- and Q-channels, respectively.

In the case of a spoofing attack, Equation (2) is rewritten as follows:

(3) $$\left\{\begin{array}{c} I_{d}=\sqrt{2P_{a}}D\left(t\right)R\left(\tau \right)\cos\left(\omega_{e}t+\theta_{e}\right)+\sqrt{2P_{s}}D\left(t\right)R\left(\tau +\Delta \tau_{s}\right)\cos\left(\left(\omega_{e}+2\pi \Delta f_{s}\right)t+\theta_{e}+\Delta \theta_{s}\right)+n_{i}\left(t\right)\\ Q_{d}=\sqrt{2P_{a}}D\left(t\right)R\left(\tau \right)\sin\left(\omega_{e}t+\theta_{e}\right)+\sqrt{2P_{s}}D\left(t\right)R\left(\tau +\Delta \tau_{s}\right)\sin\left(\left(\omega_{e}+2\pi \Delta f_{s}\right)t+\theta_{e}+\Delta \theta_{s}\right)+n_{q}\left(t\right) \end{array}\right.$$

where $P_{s}$ is the power of the spoofed satellite signal. It can be observed that the values of $I_{d}$ and $Q_{d}$ are susceptible to alteration as a consequence of spoofing attacks. Any spoofing attack will result in a modification of $I_{d}$ and $Q_{d}$. These values can be utilized for the purpose of spoofing detection.

## 3. Spoofing Detection Method

This section introduces the EDDM-MSC-MMP method, which was used to detect spoofing. The performance of the MSC-MMP in edge detection for eye diagrams was analyzed, and the methods of identifying spoofing signals and calculating thresholds were discussed.

### 3.1. EDDM-MSC-MMP

**Figure 1:**
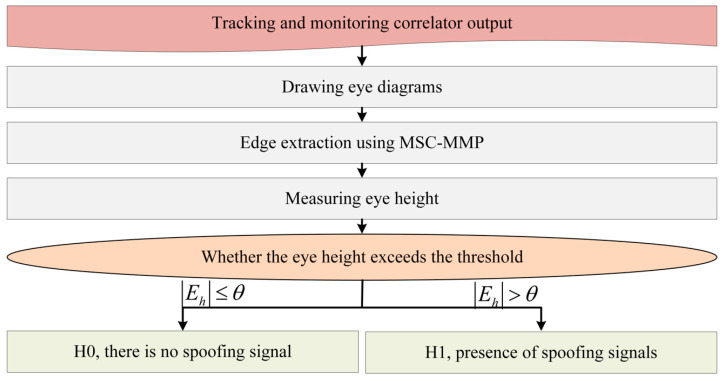
EDDM-MSC-MMP detection process.

During the signal tracking phase, the receiver stripped the satellite signal carrier. Subsequently, in the EDDM-MSC-MMP workflow, the system initially acquired the output values of the correlator. The acquired output values were then used to construct an eye diagram, in which the height of the eye indicates the signal power. Then, the MSC-MMP algorithm was applied to extract the edges of the eye diagram. Finally, the EDDM method was employed to determine whether signal spoofing exists. In this process, EDDM identified the maximum connected domain and calculated its eye height, dynamically comparing these time-varying data with a pre-set threshold to confirm the presence of spoofing. The EDDM-MSC-MMP detection process is described in Figure 1.

### 3.2. MSC-MMP

MSC-MMP is of paramount importance throughout the process of the EDDM-MSC-MMP, as it determines the algorithm’s capacity to extract the edge of the eye diagram.

**Figure 2:**
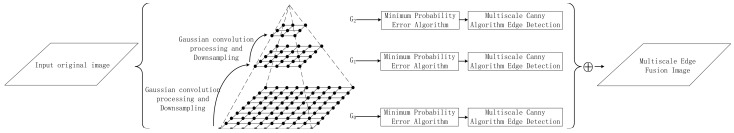
MSC-MMP algorithm flowchart.

Among edge detection algorithms, the traditional Canny algorithm often performs inadequately when extracting detailed edge information from eye diagrams [28]. This deficiency of the method results in a reduction in the accuracy of the eye diagram analysis. As the direct application of the Canny algorithm for spoof signal detection may be unreliable, this study presents a multiscale Canny algorithm based on the minimum misjudgment probability. The proposed algorithm addressed the challenge of capturing the fine texture of edges and accurately positioning edges when a large Gaussian window is used. It also avoided retaining an excessive number of texture edges with a small-scale window, markedly increasing the precision of edge detection in the eye diagram. This enabled the subsequent calculations of eye height and the identification of potential spoofing signals. The MSC-MMP algorithm (Figure 2) initially constructed a three-layer image pyramid using the original image. Subsequently, Canny edge detection was performed on each layer of the image, and the threshold with the minimum misjudgment probability was calculated for these three images of varying scales to obtain three edge images. Ultimately, by fusing these three edge images, a precise and comprehensive edge result was obtained. 

The initial image is denoted as $G_{0}$, which was set as the bottom layer (layer 0) of the Gaussian pyramid. When layer i in the Gaussian pyramid was created, Gaussian convolution was first performed on the image in layer i−1. This was followed by a downsampling step, which generated an image with a lower resolution. In this process, the height and width of the new image were halved; that is, the image dimensions were half those of the previous layer image.

(4) $$G_{i}\left(x,y\right)=\sum_{j=-2}^{2}\sum_{k=-2}^{2}F\left(j,k\right)G_{i-1}\left(2x+j,2y+n\right)$$

where $G_{i}\left(x,y\right)$ represents the image in layer i, and $F\left(j,k\right)$ denotes the 5×5 kernel function, which exhibits low-pass characteristics.

Once the three-layer Gaussian pyramid images, designated $G_{0}\left(x,y\right)$, $G_{1}\left(x,y\right)$, and $G_{2}\left(x,y\right)$, were obtained, the minimum misjudgment probability algorithm was employed to ascertain the optimal high threshold (Th) of the Canny operator. It was assumed that the probability density function of the pixels in the foreground of the image is f(x), the probability density function of the pixels in the background is b(x), and the foreground and background pixels account for $\phi_{1}$ and $\phi_{2}$ of the total pixels in the image, respectively. The probability that a foreground pixel is misclassified as background is

(5) $$E_{\phi_{1}}\left(T\right)=\phi_{1}\int_{T}^{\infty }f\left(x\right)dx$$

where T represents the assumed segmentation threshold. The probability of a background pixel being misclassified as foreground is

(6) $$E_{\phi_{2}}\left(T\right)=\phi_{2}\int_{-\infty }^{T}b\left(x\right)dx$$

The overall error segmentation probability resulting from the application of threshold T is

(7) $$E_{\phi }\left(T\right)=\phi_{1}\int_{T}^{\infty }f\left(x\right)dx+\phi_{2}\int_{-\infty }^{T}b\left(x\right)dx$$

To obtain the minimum error segmentation probability $E_{\phi }\left(T\right)$, an iterative method was employed to identify the optimal segmentation threshold T. The following steps are used:

(a) Estimate the image threshold *T*
  (8) $$T=\frac{M_{\max}+M_{\min}}{2}$$
  where $M_{\max}$ and $M_{\min}$ are the maximum and minimum gray levels of the image.
(b) Split the image to obtain two sets of pixels.
  (9) $$\varphi \left(x,y\right)=\left\{\begin{array}{c} 1,\;\;\;G_{i}\left(x,y\right)>T\\ 0,\;\;\;G_{i}\left(x,y\right)\leq T \end{array}\right.$$
  where $\varphi_{1}$ is the set of all pixels with a grayscale value greater than T and where $\varphi_{2}$ is the set of all pixels with values less than or equal to T.
(c) Calculate the average gray values $m_{1}$ and $m_{2}$ for all the pixels in $\varphi_{1}$ and $\varphi_{2}$
  (10) $$m=AVERAGE\left\{\varphi \left(x,y\right)\right\}$$
(d) Calculate the new threshold T
  (11) $$T=\frac{m_{1}+m_{2}}{2}$$
(e) Repeat steps b to d, iterating T until it is less than the custom parameter ΔT, which is set to 0.1 in this example. This value was obtained empirically through experimentation and provided the necessary accuracy without excessive calculation.
(f) Normalize the threshold
  (12) $$T_{0}=\frac{T-1}{255}$$
  where $T_{0}$ represents the calculated optimal high threshold (Th) for the Canny algorithm. In general, the ratio of the high threshold to the low threshold for the Canny algorithm is 2:1; therefore, the algorithm utilizes a low threshold (TL) of 0.5$T_{0}$.

Once the above steps have been completed, Canny edge detection was performed on images $G_{0}\left(x,y\right)$, $G_{1}\left(x,y\right)$, and $G_{2}\left(x,y\right)$ of the three-layer Gaussian pyramid. This process yields three edge images, $g_{0}\left(x,y\right)$, $g_{1}\left(x,y\right)$, and $g_{2}\left(x,y\right)$, which represent different scales. To obtain an integrated overall image, the cross-scale addition of the edge images from the three aforementioned sources was performed. Specifically, the small-scale image was resized to match the large-scale image via linear interpolation. A pixel-to-pixel addition operation was subsequently applied. In particular, the zeroth-layer image $g_{0}\left(x,y\right)$ was taken as the reference, with the first-layer image $g_{1}\left(x,y\right)$ and the second-layer image $g_{2}\left(x,y\right)$ both interpolated and normalized to the size of $g_{0}\left(x,y\right)$. Ultimately, the cross-scale addition method was employed to effectively fuse the edge details and contour information of the images at different scales, thereby enhancing the overall image quality.

(13) $$F\left(x,y\right)=g_{0}\left(x,y\right)⊕g_{1}\left(x,y\right)⊕g_{2}\left(x,y\right)$$

where $F\left(x,y\right)$ represents the image after cross-size fusion and where ⊕ represents cross-size addition.

### 3.3. MSC-MMP Performance Analysis

To verify the performance and advantages of the MSC-MMP method proposed in this paper in terms of edge detection, an eye diagram dataset was constructed, containing 100 images. The dataset includes eye diagrams of single and multiple signals at different powers, as well as similar and different signal superpositions. Morphological preprocessing and edge labeling steps were performed on these images to obtain the corresponding edge labels.

**Figure 3:**
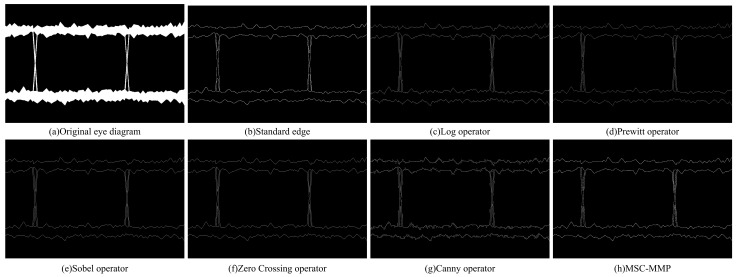
Edge detection results of various methods for an eye diagram, including Log, Prewitt, Sobel, Zero crossing, Canny, and MSC-MMP algorithm. The edges are correctly delineated and continuous, and the absence of noise indicates a favorable outcome.

We conducted comparative experiments using five commonly used edge detection algorithms and the MSC-MMP algorithm (Figure 3). The preprocessed eye diagram (Figure 3a) was employed as the original image for edge detection. The MSC-MMP algorithm not only preserved the integrity of the eye diagram edges but also mitigated the interference of textural edges. The detected edges presented the fewest discontinuities and no extraneous noise.

To assess the efficacy of the edge detection process more accurately, the optimal dataset scale (ODS), a commonly employed metric in edge extraction, was utilized as the primary evaluation criterion. The optimal dataset scale precision (ODS-P) is a metric that quantifies the mean precision of an algorithm. It represents the proportion of positive samples whose predicted results are true positive samples, which emphasizes the accuracy of the prediction. The optimal dataset scale recall (ODS-R) refers to the proportion of items that are correctly identified as positive samples among all true positive samples, thereby emphasizing the algorithm’s coverage of true positive samples. The optimal dataset scale F score (ODS-F) is a performance measure that considers both precision and recall. It is a comprehensive metric for evaluating overall performance and is expressed as follows:

(14) $$ODS-F=\frac{2\left(ODS-P\cdot ODS-R\right)}{ODS-P+ODS-R}$$

The efficacy of the generated eye diagram dataset was evaluated, and the findings are presented in Table 1. The results indicate that, among all the tested algorithms, including Log, Zero Crossing, Sobel, Prewitt, and Canny, the accuracy of each exceeded 99.8%, indicating that these algorithms demonstrate minimal error in edge recognition and rarely misidentify non-edge points as edge points. This result is primarily due to the fact that the images used in the experiment were generated using receiver correlation values, resulting in minimal noise. Moreover, the Log, Zero Crossing, Sobel, Prewitt, and Canny algorithms are exceptionally sensitive to edge transitions by design, which enables them to effectively avoid false positives during the edge detection process, resulting in very high ODS-P scores. However, the recall rates of the Log, Zero Crossing, Sobel, Prewitt, and Canny algorithms are relatively low. This is because the Log and Zero Crossing algorithms tend to miss regions with smooth or blurred edge transitions when processing the fine details of the eye diagram. The Sobel and Prewitt algorithms are prone to losing some true edges when the edge strength is weak. While the Canny algorithm effectively suppresses noise and detects prominent edges, its ability to recognize more subtle edge transitions is weaker, as it tends to focus more on the more distinct edges. In contrast, the MSC-MMP algorithm uses a multiscale edge image fusion strategy. Although it sacrifices some precision to detect more genuine edges, it can detect more edge information across different scales, successfully identifying more true edges and improving the recall rate, achieving an ODS-R of 95.4%. 

In terms of the ODS-F score, which combines precision and recall, the MSC-MMP algorithm demonstrated the optimal balance between precision and recall, with a score of 0.970. The Canny algorithm achieved a F score of 0.898, whereas the Log and Zero Crossing algorithms both had a score of 0.852. Due to their lower recall rates, these algorithms were less effective overall compared to the MSC-MMP algorithm. Overall, the MSC-MMP algorithm performed best across the three key performance metrics and was therefore chosen for edge detection in eye diagrams.

### 3.4. Threshold and Spoofing Judgment

In the context of satellite navigation signals, binary hypothesis theory was employed to detect spoofing [29]. The null hypothesis ($H_{0}$) posits that there is no spoofing signal, whereas the alternative hypothesis ($H_{1}$) asserts that there is such a signal. A comparison was made between the height of the eye diagram $E_{h}$ and the threshold θ to determine whether spoofing is present. In accordance with Equation (3), the correlation result is influenced by the power of the real signal and the noise in the absence of spoofing. As previously stated, the correlation value obeys a Gaussian distribution. Under the null hypothesis $H_{0}$, the mean value of the correlation value is μ, and the variance is $\sigma^{2}$. Consequently, the false alarm rate $P_{fa}$ is related to the threshold θ as follows:

(15) $$\begin{array}{l} P_{fa}=\rho (\left|E_{h}\right|>\left.\theta \right|H_{0})\\ =\int_{\theta }^{\infty }\frac{1}{\sqrt{2\pi }\delta }e^{-\frac{{\left(x-\mu \right)}^{2}}{2\delta^{2}}}dx \end{array}$$

when $t=\frac{x-\mu }{\delta }$, Equation (18) becomes

(16) $$\begin{array}{l} P_{fa}=\rho (\left|E_{h}\right|>\left.\theta \right|H_{0})\\ =\int_{\frac{\theta -\mu }{\delta }}^{\infty }\frac{1}{\sqrt{2\pi }}e^{-\frac{t^{2}}{2}}dt\\ =Q\left(\frac{\theta -\mu }{\delta }\right)\\ =\frac{1}{2}erfc\left(\frac{\theta -\mu }{\sqrt{2}\delta }\right) \end{array}$$

where $Q(x)=\int_{x}^{\infty }\frac{1}{\sqrt{2\pi }}e^{-\frac{t^{2}}{2}}dt$ is the right-tail function of the standard normal distribution. $erfc(x)=\frac{2}{\sqrt{\pi }}\int_{x}^{\infty }e^{-t^{2}}dt$ is the complementary error function. The expression for the threshold θ can be obtained as

(17) $$\theta =\sqrt{2}\sigma erfc^{-1}(2P_{fa})+\mu$$

Consequently, the corresponding threshold can be calculated from the set false alarm rate. In the context of spoofing detection, the objective is to determine whether a spoofing signal is present by comparing the value measured by the metric (with the eye diagram measurement $E_{h}$ serving as an illustrative example) with its threshold B. This can be expressed as follows:

(18) $$H=\left\{\begin{array}{c} \;\;\;H_{1}\;,\;\;\;\left|E_{h}\right|>\theta \\ \;\;\;H_{0}\;,\;\;\mathrm{others} \end{array}\right.$$

If $E_{h}$ exceeds θ, it is determined that there is a spoofing signal, and, otherwise, no spoofing signal is found.

## 4. Detection Coverage Simulation Experiment

Detection coverage is a key metric for evaluating the defensive efficacy of methods against various deception techniques. This section offers a detailed introduction to the commonly used CN0 and SQM (Delta, Ratio, Slope, ELP, and the combined metrics Delta+Slope and Ratio+ELP) detection methods, comparing them experimentally with the EDDM-MSC-MMP method proposed in this study. It presents simulation results of detection coverage for different methods when dealing with various spoofed signals, which vary in power, code phase offsets, and carrier offsets.

The L1 signal of the GPS 8 satellite was employed to conduct a spoofing simulation. A total of 4,410 grid experiments were conducted, each lasting 10 seconds, with spoofing occurring for a period of 5 to 10 seconds. The receiver employed was a GNSS software-defined radio. The signal simulation parameters were set as follows: the range of the relative power advantage of the spoofing signal was 1 dB to 10 dB, with a step size of 1 dB; the range of the code phase offset $\Delta \tau_{s}$ was 0 to 1 chip, with a step size of 0.05 chips; and the carrier phase offset $\Delta \theta_{s}$ ranged from 0 to 2π. The step size for the spoofing signal was 0.1π, resulting in a noise level comparable to that of the real signal. The false alarm rate was $P_{f}\leq 0.01$. All other parameters of the spoofing signal were identical to those of the real signal. To ensure the timely detection of spoofing, the predetection integration time was $T_{d}=0.005s$. One spoofing decision was made per millisecond, and the detection rate was calculated after each experiment. The rate of detection is defined as follows:

(19) $$P_{d}=\frac{\mathrm{Time}\left\{\mathrm{Measurements}\;\mathrm{exceed}\;\mathrm{the}\;\mathrm{threshold}\right\}}{\mathrm{Time}\left\{\mathrm{Presence}\;\mathrm{of}\;\mathrm{spoofing}\;\mathrm{signal}\right\}}$$

This is the ratio of the time during which the metric measurement exceeds the threshold (the time during which spoofing is detected) to the total time during which the spoofing signal is present.

**Figure 4:**
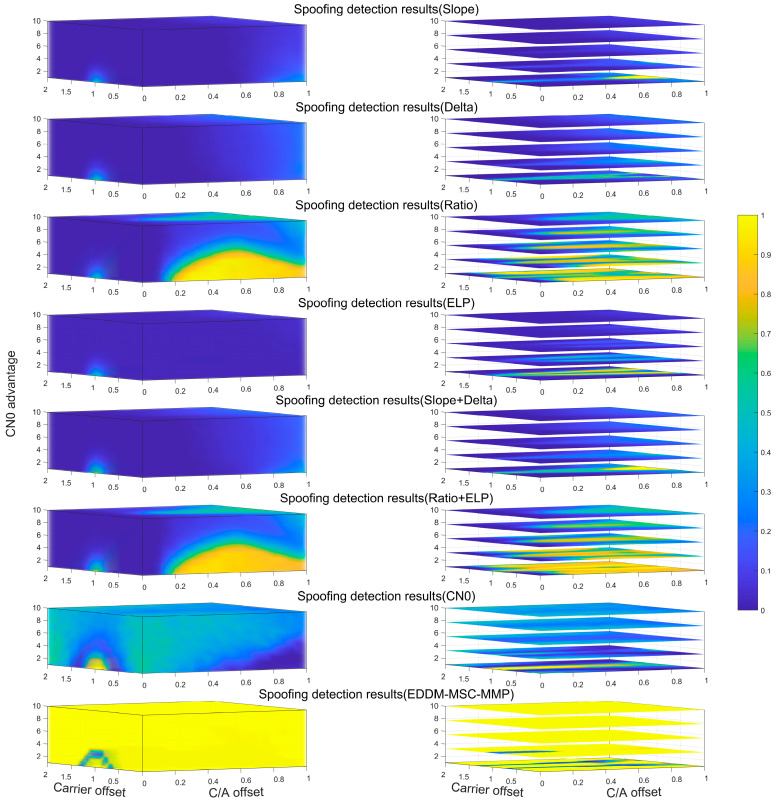
Detection rates with respect to the possible relative power advantages, code phases, and carrier phases of the spoofing signal ($C/N_{0}=$1 to 10 dB, $\Delta \tau_{s}=$0 to 1 chip, and $\Delta \theta_{s}=$0 to 2π rad) with $T_{d}=1s$. From top to bottom, the rows of the subplots correspond to $C/N_{0}$, ELP, Delta, Ratio, Slope, Slope+Delta, Ratio+ELP, and the EDDM-MSC-MMP. The light yellow indicates a detection rate of 100%, whereas the dark blue indicates a detection rate of 0%.

The detection results are shown in Figure 4. The first column shows a three-dimensional representation of the detection results, and the second column shows the corresponding slices. The X-axis denotes the relative code phase offset of the spoofing signal, the Y-axis indicates the relative carrier phase offset of the spoofing signal, and the Z-axis denotes the relative power advantage of the spoofing signal. The color of the grid indicates the detection rate. As illustrated in the figure, the two SQM metrics, Delta and Slope, exhibit a notable degree of correlation in their detection rates. Their detection rates are low because both metrics perform spoofing detection on the basis of unilateral information about the relevant peak, which results in a relatively low level of accuracy. In contrast, the ELP and Ratio metrics adopt a complementary approach that utilizes bilateral information from the relevant peaks, thereby improving the detection performance to a certain extent. While the SQM metric combination exhibits better detection performance than the use of a single metric, this improvement is constrained by the inherent limitations of the original single metric. In particular, the combination of Delta and Slope does not yield a significant improvement in the detection rate, and the combination of Ratio and ELP also results in only very limited performance gains. The CN0-based detection method has poor detection results because spoofing signals have the same noise characteristics as the real signal, and, even though spoofing signals have a relative power advantage, the $C/N_{0}$ change in the receiver is not obvious. In contrast, the overall detection rate of the EDDM-MSC-MMP is significantly greater. This is because the EDDM-MSC-MMP employs a strategy of real-time monitoring of amplitude changes in the correlation value following the receiver integration–cancelation process. This effectively blocks the influence of the noise power and ensures that the sensitivity of spoofing detection is maintained.

Furthermore, as the relative power of the spoofing signal increases, the detection rate of the SQM metric gradually decreases. This is primarily because, when the spoofing signal is very similar to the authentic signal and has considerable power, the spoofing signal will rapidly influence the receiver. The receiver’s phase-locked loop quickly disengages from the authentic signal and locks onto the spoofing signal, resulting in a modification of the duration of the slope change in the receiver’s correlation peaks. The greater the similarity of the spoofing signal and the higher the relative power advantage, the faster the intrusion, and the shorter the duration of the abnormal correlation peak slope. Since the SQM metric relies heavily on changes in the correlation peak slope, the decrease in anomaly duration ultimately leads to a decrease in the detection rate. The CN0-based detection method is less affected, and the detection rate basically remains between 30% and 40%. The EDDM-MSC-MMP method proposed in this article is not affected by this, and, except for the lower detection rate under the conditions of lower relative power advantage, $\Delta \theta_{s}=0.7\pi$ and $\Delta \theta_{s}=1.3\pi$, it maintains a detection rate of over 95%.

To conduct a more objective evaluation of the performance of the detection method, a new metric, called ‘detection coverage’, is established. In contrast to the detection rate, the detection coverage provides insight into the ratio of the detectable volume to the total volume within a specified detection area. This ratio can comprehensively reflect the detection method’s capacity to identify spoofing signals with varying relative power advantages, code phase delays, and carrier phase delays. A minimum acceptable detection rate, designated $P_{\min}$, is established. If $P_{d}>P_{\min}$, the area is classified as detectable; otherwise, it is designated as undetectable. In our experimental setup, the detection area for each experiment was 1 unit of volume, and the entire experimental volume comprised 4410 units. The detection coverage was defined as follows:

(20) $$\mathrm{Detection}\;\mathrm{coverage}=\frac{\mathrm{Volume}\;\mathrm{of}\;\mathrm{detectable}\;\mathrm{area}}{\mathrm{Total}\;\mathrm{volume}}$$

**Figure 5:**
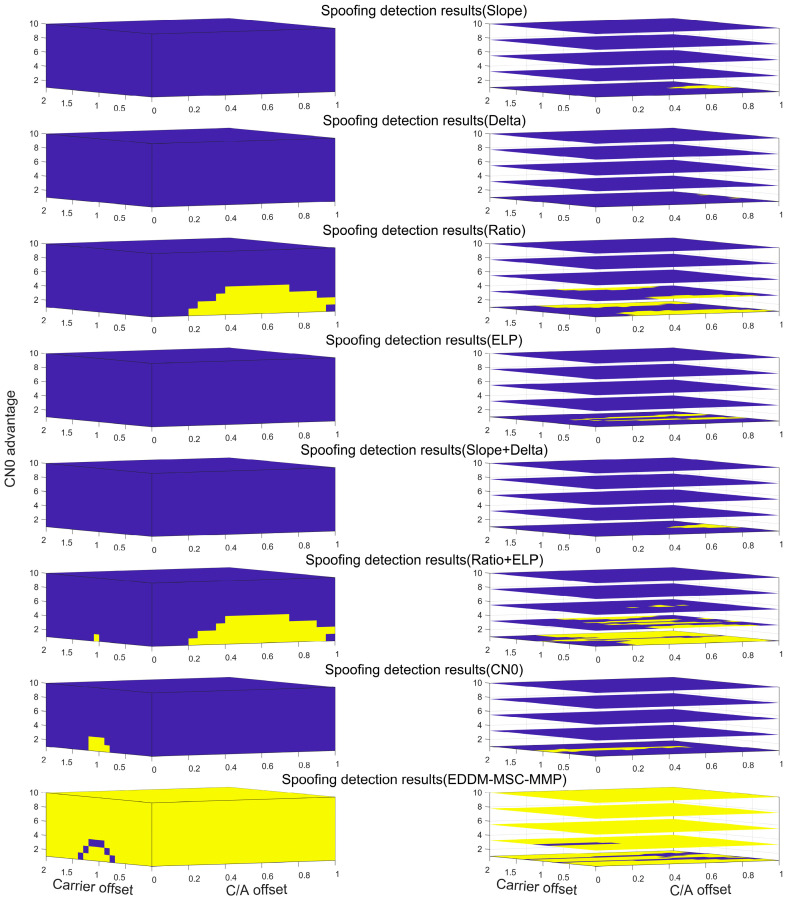
Detectable areas of the various detection methods at $P_{\min}=80\%$. From top to bottom, the rows of the subplots correspond to $C/N_{0}$, ELP, Delta, Ratio, Slope, Slope+Delta, Ratio+ELP, and the EDDM-MSC-MMP. The yellow indicates detectable areas, while the dark blue indicates undetectable areas.

The result for $P_{\min}=80\%$ is presented (Figure 5). The yellow indicates detectable areas, while the dark blue indicates undetectable areas. The measurement of the detection coverage enables the effective evaluation and optimization of the overall performance of the detection system. A statistical analysis was conducted to determine the coverage of each detection method on the basis of varying minimum acceptable detection probabilities (Table 2). Regardless of whether SQM single-metric or SQM composite-metric detection is employed, the high similarity of spoofing signals results in a detection coverage of less than 10% when $P_{\min}>90\%$, which is considered inadequate. No significant distinction can be observed between the various SQM metrics. Only when $P_{\min}<50\%$ does the number of detectable regions increase for some SQM metrics, such as Ratio and Ratio+ELP. None of the CN0-based spoofing detection methods demonstrate optimal performance when $P_{\min}\geq 50\%$. Instead, there is only a limited increase in the number of detectable regions until $P_{\min}<30\%$. In contrast, the EDDM-MSC-MMP method proposed in this paper achieves a detection coverage rate of 95.35% even when $P_{\min}=95\%$, which is excellent detection performance. We provide a summary of the detection coverage of the eight detection methods at varying minimum acceptable probabilities. The comprehensive performance is ranked from high to low as follows: EDDM-MSC-MMP > Ratio+ELP > Ratio > CN0 > ELP > Slope+Delta > Delta > Slope. The MSC-MMP method proposed in this paper exhibits superior detection performance to that of other methods in terms of spoofing detection across diverse scenarios, including varying powers, code phase offsets, and carrier offsets.

## 5. Experimental Results on the TEXBAT Dataset

This section presents an evaluation of various spoofing detection methods using a publicly accessible dataset provided by the University of Texas at Austin. The dataset includes eight authentic spoofing instances. We have conducted a comprehensive performance verification of different detection methods. Given the constraints of article length, we selected Cases 1, 2, and 3 as representative examples for comparison and evaluated the ROC curve performance of each method. 

### 5.1. Receiver Operating Characteristic Curve Experiment

**Figure 6:**
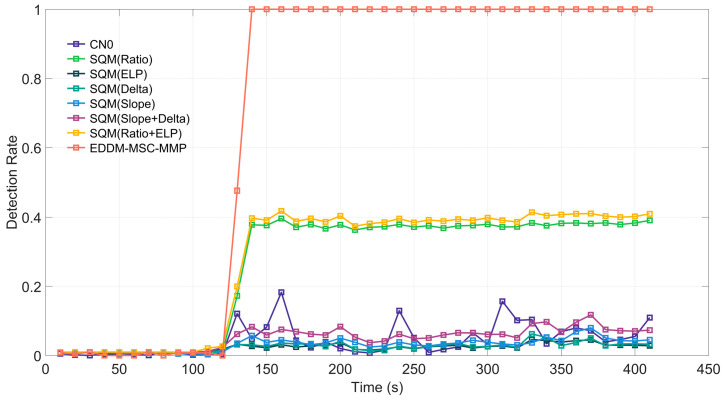
Detection rate of each detection method for Case 1 of the TEXBAT dataset.

Each of the cases (1, 2 and 3) was tested individually for a total duration of 410 seconds. In these cases, spoofing attacks typically occurred between the 90th and 120th seconds and persisted until the conclusion of the signal. To assess the efficacy of time-domain transient detection more accurately, we calculated the detection rate at 10-second intervals. We established a false alarm rate $P_{f}\leq 0.01$ and a predetection integration time $T_{d}=100\;ms$. Case 1 in TEXBAT involves a scenario in which the attacker first blocks the real signal and then quickly switches to a spoofing signal that is weaker than the real signal. The receiver platform is static. Although the power of this spoofing signal is weaker than that of the real signal, it never coexists with the real signal. The results of the detection process are illustrated in Figure 6. During the first 120 s, the spoofing signal is not activated, and the detection rates of all methods are less than the false alarm rate of 0.01 that was established. 

When the spoofing signal is subsequently activated, the curves of each detection method begin to change. In particular, after the spoofing signal begins, the instantaneous detection rate of the EDDM-MSC-MMP exhibits a pronounced increase, reaching a sustained 100% detection rate after 140 seconds. Interestingly, the curves (e.g., Slope, Delta, ELP) of the detection methods that rely only on the change in correlation peaks in the I-channel lag significantly behind the other curves, with a detection rate of less than 10%; this is because Case 1 is a spoofing-switching case, where there is no overlap between the spoofing signals and real signals and where the correlation peaks change very little when the spoofing signal arrives. Although the Ratio method also performs detection on the basis of the correlation peak changes, it utilizes both in-phase and quadrature signals for detection, and the detection performance is better than the Slope, Delta, and ELP methods. The combined detection methods are all better than the single-metric detection methods, but their performance improvement is limited. The CN0-based detection method also has a low detection rate because of the small change in CN0 in Case 1.

**Figure 7:**
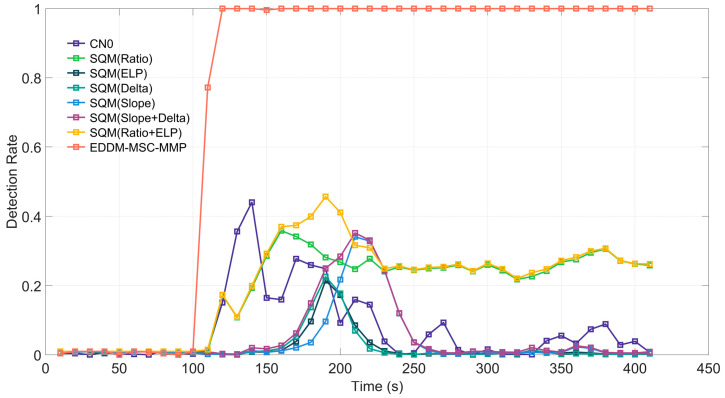
Detection rate of each detection method for Case 2 of the TEXBAT dataset.

In TEXBAT Case 2, the spoofing signal is 10 dB higher in power than the real signal. Additionally, the phase difference between the carrier of the spoofing signal and the real signal is not maintained at a fixed value, and the receiver platform is stationary. Figure 7 illustrates the efficacy of each detection method in Case 2. In this case, the spoofing signal is superimposed from the 100th second onward. In contrast to Case 1, the detection methods have distinct detection rates, as well as varying response speeds and detection rate. Among the methods, the EDDM-MSC-MMP has the fastest response time, followed by CN0, Ratio+ELP, and Ratio. The response times of the ELP, Delta, Slope, and Delta+Slope methods are quite similar. 

With respect to the detection rate, following the detection of the spoofing signal, the detection rate curves of the methods exhibit a peak shape, except for that of the EDDM-MSC-MMP; this method maintains a high detection rate of 100%. The curves of the other methods are characterized by an initial increase in the detection rate, followed by a decrease. The maximum detection rate achieved by these methods does not exceed 50%. The reason for the difference between the curves in Case 2 and Case 1 is that, in Case 2, the spoofing signal coexists with the real signal, and the greater power of the spoofing signal enables it to suppress the real signal. Because their carriers are out of sync, the receiver is unable to immediately and completely lock onto the spoofing signal, which causes a small distortion in the correlation peak function over a short period. Therefore, although the method based on the correlation peak function shows some detection rates, its overall detection performance is not satisfactory.

The distinction between Case 3 and Case 2 lies in the utilization of a matched-power scheme, in which the power of the spoofing signal decreases from 10 dB to 1.3 dB, whereas the phase difference between the carrier of the spoofing signal and the real signal remains unaltered.

**Figure 8:**
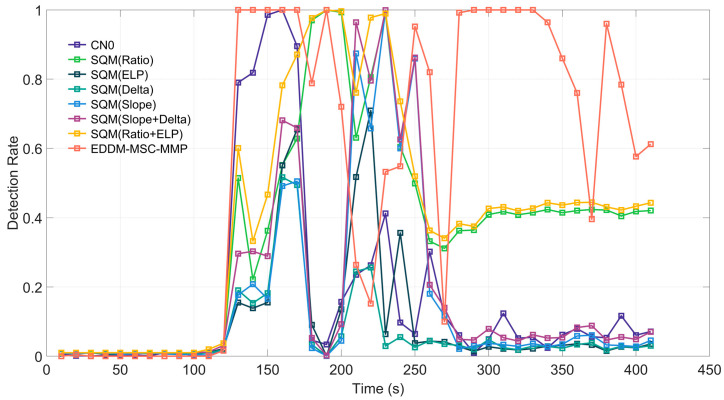
Detection rate of each detection method for Case 3 of the TEXBAT dataset.

In Case 3, a notable increase in detection efficacy is observed when the correlation peak function and the CN0 method are employed (Figure 8). This improvement can be attributed to the reduction in the power advantage of the spoofing signal in Case 3 to 1.3 dB, coupled with a fixed phase difference with the real signal. This increases the time required for the receiver’s phase-locked loop to track the spoofing signal, resulting in an increase in the distortion time of the correlation peak function. As a result, the correlation peak detection method can more easily identify spoofing signals. In Case 3, the curves of all detection methods showed significant fluctuations. After the spoofing signal intrusion, the detection rates of CN0, Slope, Delta, ELP, and Slope+Delta rapidly decreased to nearly zero. In contrast, the EDDM-MSC-MMP, Ratio, and Ratio+ELP were able to maintain a certain detection rate.

To conduct a comprehensive evaluation of the performance of the various detection methods, we calculated and simulated the ROC curves for Cases 1, 2, and 3. 

**Figure 9:**
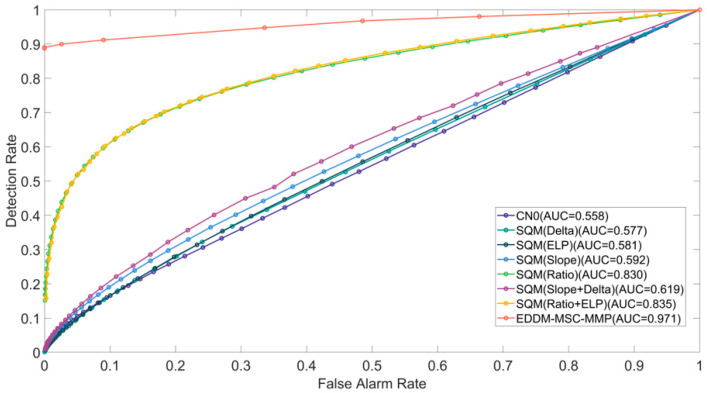
ROC curves for the detection methods in Case 1 of the TEXBAT dataset. A larger area under the ROC curves indicates better performance.

**Figure 10:**
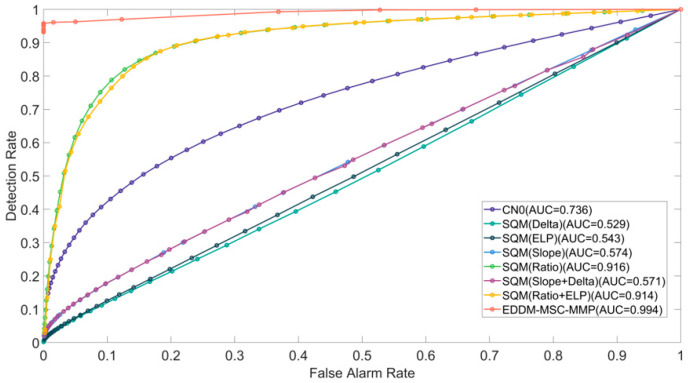
ROC curves for the detection methods in Case 2 of the TEXBAT dataset.

**Figure 11:**
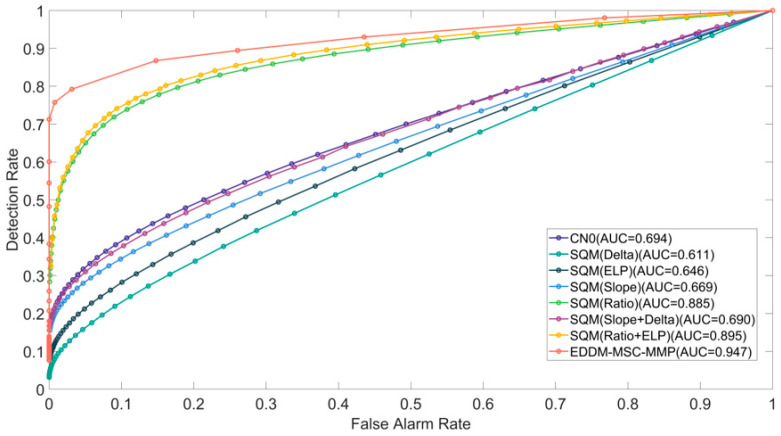
ROC curves for the detection methods in Case 3 of the TEXBAT dataset.

During the interval of 0 to 410 s designated for spoofing detection, the corresponding detection rate $P_{d}$ and false alarm rate $P_{f}$ were calculated by adjusting the threshold of the detection method, and the ROC curve was plotted. The ROC curves for Cases 1, 2, and 3 are presented in Figure 9, Figure 10 and Figure 11, respectively. To evaluate the detection performance, we conducted a further analysis of the area under curve (AUC) for the receiver operating characteristic (ROC) curves, which has a theoretical maximum value of 1. The AUC value of the EDDM-MSC-MMP method is markedly higher than those of the other methods, exceeding 0.94 in all cases. Notably, it reaches 0.994 in Case 2, approaching the ideal value of 1. In comparison, the AUC values of the alternative detection methods range from 0.65 to 0.9. The combination of these metrics yields the following performance ranking from high to low: EDDM-MSC-MMP > Ratio+ELP > Ratio > CN0 > Slope+Delta > ELP > Slope > Delta. In conclusion, the EDDM-MSC-MMP achieves a better ROC curve performance than the other detection methods in all the cases, including cases of suppressed spoofing signals, highly similar spoofing signals, and spoofing signal switching.

### 5.2. Comprehensive Performance Verification with TEXBAT Dataset

We counted the detection rates of each detection method for the eight cases in the TEXBAT dataset (Table 3). In the experiment, the period from 110 s to 410 s (300 s in total) was selected as the period in which the spoofing signal coexisted with the real satellite signal and was in the spoofing phase, $T_{d}=100\;ms$, and $P_{f}\leq 0.01$.

We have already discussed Cases 1 to 3, so we will not repeat the details here. In Case 1, the EDDM-MSC-MMP achieved a detection rate of 95.23%, which was significantly better than those of the other methods. Ratio+ELP had a detection rate of 29.75%, and Ratio had a rate of 28.77%, while Slope, Delta, ELP, Slope+Delta and CN0 all had detection rates below 10%. This result clearly shows that the EDDM-MSC-MMP had the best detection performance in Case 1. In Case 2, the detection rate of the EDDM-MSC-MMP increased to 99.31%, and the remaining methods remained inefficient, demonstrating the superior performance of the EDDM-MSC-MMP. In Case 3, although the detection rate of the EDDM-MSC-MMP decreased slightly, it still achieved the highest detection rate, 80.71%, among the correlation function-based methods. 

In Case 4, compared with Case 3, the power advantage of the spoofing signal is reduced from 1.2 dB to 0.4 dB, accompanied by a position offset. In this scenario, the methods with a detection rate above 50% included the EDDM-MSC-MMP, Ratio+ELP, and Ratio. The EDDM-MSC-MMP ranked first with a detection rate of 72.90%, followed by Ratio+ELP with 69.06% and Ratio with 51.70%. The scenario in Case 5 is similar to that in Case 2, except that the receiver platform is dynamic. The dynamic receiver platform and the intervention of spoofing signals had a significant effect on the detection method based on correlation peaks, causing its detection ability to drop sharply, whereas there was almost no effect on the other methods. The EDDM-MSC-MMP maintained a high detection rate of 98.11%. The scenario in Case 6 is similar to that in Case 4, except that the receiver platform is dynamic, which further increases the difficulty of detection. Even in this more complex environment, the detection rate of the EDDM-MSC-MMP was 21.60%, which was significantly better than those of the competing methods.

Finally, Case 7 implements carrier phase alignment on the basis of Case 3, and Case 8 introduces zero-delay secure code estimation and replay attacks on the basis of Case 3. In both cases, the detection rate of the EDDM-MSC-MMP exceeded 75%, whereas the detection rates of Slope, Ratio, Slope+Delta and Ratio+ELP were similar, all between 60% and 70%. Delta, ELP, and CN0 had poor detection performance, with detection rates less than 5%.

In summary, the EDDM-MSC-MMP demonstrated excellent performance in a variety of test scenarios. The analysis of Cases 1 to 8 shows that the EDDM-MSC-MMP can maintain an efficient detection rate with a false positive rate of less than or equal to 0.01 in scenarios with either a static or dynamic receiving platform and with different types of spoofing signals. In particular, in Case 5, even though the detection efficiency of the other methods was close to zero, the EDDM-MSC-MMP was able to achieve a detection rate of over 95%, which was significantly better. This consistently excellent performance highlights the reliability and efficiency of the EDDM-MSC-MMP in complex signal environments, which could make it the technology of choice for detecting spoofing signals.

## 6. Conclusions

In this study, we propose an MSC-MMP algorithm for extracting the edges of an eye diagram. On this basis, we propose the EDDM-MSC-MMP to detect spoofing signals. Through theoretical analysis, simulation, and experimentation, we draw the following conclusions:

1. The MSC-MMP algorithm significantly improves the accuracy of eye diagram edge detection. In particular, the algorithm effectively suppresses the interference of texture edges while maintaining the integrity of the edges. It solves the problems of the difficulty of detecting fine texture edges and inaccurate edge positioning when large Gaussian windows are used, and it avoids retaining too many texture edges when small windows are used. Tests on the eye diagram dataset that we constructed show that the MSC-MMP algorithm achieves an optimal balance between accuracy and recall, achieving an ODS-F score of 0.970, which significantly exceeds the Canny algorithm’s score of 0.898 and the Log and Zero Crossing algorithm’s score of 0.852. These results demonstrate the advantages and potential of the MSC-MMP algorithm for eye diagram edge detection.
2. The EDDM-MSC-MMP is a highly sensitive spoofing detection algorithm with high detection capability and excellent detection coverage. Among a large number of algorithms, the EDDM-MSC-MMP demonstrated the highest stability, with a detection coverage of over 95% under different minimum acceptable detection rate requirements (from 95% to 30%). Even with a minimum acceptable detection rate of 95%, the detection coverage of the EDDM-MSC-MMP still reached 95.35%, which is significantly higher than the highest coverage of 1.02% for the SQM (including the composite metrics Ratio+ELP and Slope+Delta) and 0.75% for the CN0 method, and it is clearly superior to the competing algorithms.
3. The EDDM-MSC-MMP method has an excellent ROC curve and overall performance in a realistic complex environment. On the real TEXBAT spoofing dataset, its AUC value was significantly higher than those of the other detection techniques, reaching 0.971, 0.994, and 0.947 in three typical cases. In contrast, the AUC values of the other methods ranged from 0.65 to 0.92. In the eight cases in the dataset, the EDDM-MSC-MMP consistently achieved the highest detection results, outperforming the other algorithms by 5% to 98%. In particular, the EDDM-MSC-MMP was able to achieve a detection rate of up to 98.11% even when the other methods performed poorly, as in Case 5. This consistent outstanding performance highlights the reliability and efficiency of this method in complex signal environments.

## Figures and Tables

**Table 1:** Statistics for edge detection results.

Algorithm	ODS-P	ODS-R	ODS-F
Log	99.9%	74.3%	0.852
Prewitt	99.9%	67.5%	0.806
Sobel	99.9%	66.9%	0.801
Zero Crossing	99.9%	74.3%	0.852
Canny	99.8%	81.6%	0.898
MSC-MMP	98.7%	95.4%	0.970

**Table 2:** Coverage of various detection methods.

Detection Method	Minimum Acceptable Detection Rate							
	95%	90%	80%	70%	60%	50%	40%	30%
Slope	0.98%	0.98%	1.02%	1.09%	1.09%	1.29%	1.63%	2.77%
Delta	0.00%	0.00%	0.05%	0.11%	0.41%	0.88%	2.34%	4.13%
Ratio	0.00%	2.29%	10.18%	19.50%	29.52%	38.05%	45.49%	53.06%
ELP	0.18%	0.45%	1.63%	2.72%	3.72%	5.51%	7.05%	9.82%
Slope+Delta	1.02%	1.07%	1.09%	1.27%	1.84%	2.95%	4.10%	7.35%
Ratio+ELP	0.86%	5.03%	18.25%	28.16%	34.76%	42.70%	49.43%	56.33%
CN0	0.75%	0.95%	1.75%	2.34%	3.17%	6.37%	22.93%	50.25%
EDDM-MSC-MMP	95.35%	95.94%	96.37%	96.67%	96.96%	97.10%	97.32%	97.57%

**Table 3:** Detection rates of different methods on the TEXBAT dataset.

Detection Method	TEXBAT Dataset							
	Case 1	Case 2	Case 3	Case 4	Case 5	Case 6	Case 7	Case 8
Slope	2.89%	4.27%	19.99%	6.60%	0.22%	1.17%	62.11%	62.40%
Delta	0.78%	0.92%	5.44%	20.37%	0.08%	1.19%	0.93%	2.76%
Ratio	28.77%	14.12%	43.32%	51.70%	0.01%	1.15%	66.70%	66.84%
ELP	1.33%	1.46%	10.50%	42.87%	0.29%	2.67%	2.16%	2.59%
Slope+Delta	3.53%	4.77%	22.42%	24.48%	0.27%	2.03%	62.20%	62.70%
Ratio+ELP	29.75%	15.47%	47.70%	69.06%	0.30%	3.67%	67.06%	67.28%
CN0	5.98%	10.18%	23.70%	45.75%	0.18%	1.72%	10.99%	16.19%
EDDM-MSC-MMP	95.23%	99.31%	80.71%	72.90%	98.11%	21.60%	79.39%	76.84%

## Data Availability

The TEXBAT datasets are provided by the radio navigation laboratory of the University of Texas at Austin and can be downloaded via the following hyperlink: https://radionavlab.ae.utexas.edu/texbat, accessed on 28 January 2025.
